# Learning Tools Using ChatGPT in the Biochemistry Class: Creating Notes and Performance on Exams

**DOI:** 10.1002/bmb.21904

**Published:** 2025-05-15

**Authors:** Ana Roman, Maria Simaitis, Kate Sheely, Yotam M. Roth, John T. Tansey, John Cogan

**Affiliations:** ^1^ The Ohio State University Columbus Ohio USA; ^2^ Department of Chemistry, Program in Biochemistry and Molecular Biology Otterbein University Westerville Ohio USA; ^3^ Department of Chemistry and Biochemistry The Ohio State University Columbus Ohio USA

**Keywords:** AI, biochemistry, ChatGPT, learning tools, undergraduate STEM education

## Abstract

ChatGPT has emerged as a popular choice in education that has transformed the experience for both teachers and students. This study investigates the performance of ChatGPT in aiding learning in the biochemistry classroom in two ways. We sought to determine how effective ChatGPT 3.5 was in generating study materials for an introductory biochemistry course. A lecture was delivered in class and transcribed. The resulting transcript was curated, then submitted to ChatGPT 3.5 to generate a summary, a set of notes, and an outline. Each artifact was verified and given to students to help prepare for a quiz. Students were asked about the use of AI‐generated materials compared to other study materials. Results were bimodal for the AI‐generated materials, with some students indicating that the materials were useful while others preferred traditional study materials such as the text or class notes. We also compared the performance of ChatGPT on open‐note biochemistry exams that students had taken. The exams were completed by ChatGPT in two modes: with and without access to external tools. Performance metrics were used to evaluate ChatGPT performance and student responses. The results showed that ChatGPT was unable to pass the exams. This research provides valuable insights into the potential of ChatGPT in educational settings, highlighting strengths and limitations. The implications of these findings may inform the design of future AI‐assisted tools and contribute to the ongoing discussions surrounding the integration of AI in education.

## 1 Introduction

Over the past 2 years, Large Language Model (LLM) artificial intelligence (AI) programs have become widely available [1]. These programs are efficient at performing natural language processing (NLP) tasks, including recognition, translation, prediction, and generation of text, but can also be trained to perform other tasks such as predicting protein structures [2] and coding. ChatGPT is based on a specific LLM model, the generative pre‐trained transformer (GPT), developed and released by OpenAI in November 2022. ChatGPT's ability to search for patterns and generalize information to generate human‐like responses has led to its utilization in various settings. Researchers such as Noam Chomsky, the father of modern linguistics, are arguing that while revolutionary, these AI generative models differ profoundly from how humans reason and use language [3]. It has been shown that ChatGPT suffers from hallucinations, seemingly producing convincing but inaccurate responses, and that on reasoning tasks its accuracy is relatively low [4].

There are advantages and disadvantages to using generative AI models such as ChatGPT in education [5]. The quick responses and the ease of use have been reported as the main drivers which promote ChatGPT as a tool that provides accessible learning for students and a time‐saving scaffold for educators. As an AI‐powered model with adaptive learning capabilities, ChatGPT is a possible asset to the constructivist theory‐based education that requires a more active and autonomous approach. The students have eagerly embraced this technology, reporting largely only positive benefits such as increased quality of work, independence, and confidence [5]. However, as observed by faculty and confirmed by research, the negative consequences that arise from overreliance, especially given the inaccuracy inherent to the model, are unique challenges to this paradigm [5, 6]. For students to use the tool, they need to be taught critical thinking skills and how to efficiently use AI chatbots in the learning process [7].

Use of notes and the taking of notes are generally regarded as fundamental aspects of higher learning [8, 9, 10]. The act of note taking keeps students engaged with the topic and thus helps with understanding and remembering the information. Research indicates that handwritten note‐taking often enhances student performance on tasks that require deeper processing and improves long‐term conceptual understanding compared to typed notes [11, 12]. This suggests that the physical act of writing by hand may engage cognitive processes more effectively, leading to better comprehension of material. While debate exists as to how to best take notes (i.e., should students be provided with outlines or the lecturer's notes to begin with or use some format such as Cornell notes) [13, 14, 15, 16], it is clear that at some level notetaking itself can be problematic [17]. Notetaking combines multiple skills including handwriting, typing or drawing, processing of audiovisual information, background knowledge, and sustained attention that puts students on an uneven playing field while content is introduced [18]. AI‐powered tools can be used to fill in those gaps by generating automated notes; for example, ChatGPT can be used to summarize text, documents and videos [19].

ChatGPT's fast adoption in education has prompted researchers and other entities to assess and analyze its expertise in numerous subjects, including medicine, law, and engineering, to name a few [20, 21, 22]. The performance has been a mixed bag, with ChatGPT being able to pass or even excel on some tests but failing others. It has been reported that while the question type (binary, MCQ, and descriptive) does not seem to affect the performance, the question difficulty does have an impact. ChatGPT has also been used to assess biochemistry and biology courses [23, 24, 25]. A medical biochemistry, undergraduate biochemistry, and glycobiology course were used to examine how ChatGPT performed on exams and rate the learning capabilities. We note that ChatGPT 3.5 has been used in these studies. While it achieved a passing grade on the exams or MCQ portions thereof, the researchers highlighted similar issues of hallucinations, lack of accuracy, and relevancy, delineating the need for improvement. As found in other domains, complex questions rated difficult by humans were as challenging for ChatGPT.

The current study aims at examining the twofold usefulness of ChatGPT in a biochemistry class: for instructors creating study materials for students and assessing biochemistry exams. First, we generated material from a lecture using ChatGPT and provided students with those notes. We then assessed the students' subjective preference for use in preparing for a traditional assessment. Second, we analyzed how ChatGPT performs on introductory biochemistry exams compared to college students. We utilize both ChatGPT 3.5 and ChatGPT 4.0, which is the current ChatGPT version that is more accurate and includes a novel image feature analysis. Thus, this research attempts to compare current AI models with the human capacity for analytical thinking, critical reasoning, and understanding the underlying principles of biochemistry. The findings may inform the development of more effective AI‐driven educational tools, ultimately enhancing teaching and assessment in science courses.

## 2 Materials and Methods

### 2.1 ChatGPT Based Study Materials

#### 2.1.1 Class and Students

This aspect of the study was conducted in Biochemistry I (BMB 4500) at Otterbein University. Otterbein is a small primarily undergraduate institution in Westerville, Ohio, in the United States. The course is the first of two in a majors' biochemistry sequence typically taken in the junior or senior year and covers both the introduction and description of biological molecules and their metabolism. It has a full year of organic chemistry as a prerequisite, and greater than 90% of the students in the class are biochemistry and molecular biology majors. The remainder are typically pre‐veterinarian, pre‐medical, or pre‐physician assistant students from biology, equine science, or allied health majors. The class that participated in this study consisted of 26 students. The class uses multiple active pedagogies to engage students but complements these with short lectures as needed. As such, this exercise did not deviate significantly from normal classroom activities.

#### 2.1.2 AI Generation of Study Materials

This study was conducted on a lecture covering aspects of fatty acid catabolism, specifically coupling and transport of fatty acids into the mitochondrial matrix and beta oxidation. The lecture was approximately 20 min in length with several pauses for student questions. This was captured using a standard microphone connected to a laptop running Microsoft Word. The dictate function of Word (found under the “Home” tab) was used to generate a transcript of the lecture. This is referred to in this work as the transcript.

Following transcription, the investigator examined the transcript for accuracy and edited it. This was done primarily to ensure that the transcription was accurate (e.g., there were no instances where the software used an incorrect term), and scientific terms were properly spelled and used. Other edits included removal of paralanguage (filler words found in speech) and removal of sidebar conversations which clarified points made in the lecture (e.g., student questions). The result of this edit is referred to in this work as the curated transcript and is approximately 1600 words.

The curated transcript was submitted to Chat‐GPT version 3.5, and it was asked to generate a summary, an outline, and a set of notes. All three tools were examined by the instructor for accuracy. The summary version of the transcript was approximately 130 words. The outline consisted of seven level one headings, each of which had two to eight subsections, and the notes were a series of 15 bullet points. The generation of each of these tools took less than a minute.

#### 2.1.3 Assessment Method

Each of the four tools generated (the transcript, the outline, the notes, and the summary) was provided to students to help them prepare for a standard weekly quiz. Students were still able to use any other study materials of their choice and were not required to use the four new tools. The day following the quiz, students were given a brief survey that asked them about their use of the AI‐generated study materials compared to other materials to which they also had access (e.g., the text, lecture notes, study guides, homework problems, and in class exercises).

### 2.2 ChatGPT Performance on Biochemistry Exam Questions

#### 2.2.1 Class and Exams

This aspect of the study was conducted using exams from Ohio State University's BIOCHEM 4511, Introduction to Biochemistry, a class of around 250 students. The course deals with the molecular basis of structure, metabolism, genetic replication, transcription, and translation in plants, animals, and microorganisms. It has one semester of organic chemistry as a prerequisite. It is a lecture‐based course where notes are provided, but students are encouraged to add on to the material provided as needed. The class has an online textbook that uses automatic grading, but solutions to problems are not available to students.

The exams used in this study were given to students in an open‐note open‐web format; students were allowed to use all notes collected, search engines, and online tools, defined as an interactive website that provides information based on input (Excel, Protein Data Bank, Expasy—Compute pI/Mw tool). ChatGPT was released during this time, so it was a novel tool at the time. There were four exams administered. Most of the questions included either images or tools. The questions that required the students to either draw or conduct all work in an external tool (e.g., Excel) were removed from the analysis to create a best‐case scenario in the ChatGPT comparison. The analysis was therefore conducted on the remaining 55 questions, of which 42 included images and 8 included tools.

Questions from these exams were then classified using Bloom's taxonomy to gauge performance on three different question types, namely remember, analyze, and apply. The other types in the Bloom's taxonomy were not utilized since they did not apply to any of the questions. The three question types used are explained in Table 1. There were 20 remember questions, 21 apply questions, and 14 analyze questions in the final set.

**TABLE 1:** Bloom's taxonomy.

Question types	Definition used	Classification of question
Remember	Recall basic facts and concepts	Information clearly stated in class or lecture notes (required rote memorization)
Analyze	Connections between two or more ideas	Two or more ideas could be clearly taken out of question, required knowledge of both to answer
Apply	Information used in a new context or situation	Context of question could not be found in class or lecture notes, but required concept from class

#### 2.2.2 ChatGPT Test Conditions

We tested ChatGPT on the four exams under the following conditions: (1) the free ChatGPT 3.5 with no additional input (ChatGPT), (2) ChatGPT 3.5 with additional input (ChatGPT+), and (3) the paid version of ChatGPT 4 with no additional input (ChatGPT 4). The input provided in each condition is described in Table 2.

**TABLE 2:** ChatGPT test conditions guidelines.

Conditions	Guidelines
ChatGPT	The questions were entered in ChatGPT 3.5 as appeared on the exam. No interpretations were made, and no external tools were used.
ChatGPT+	External information used to modify the questions before using ChatGPT 3.5: (1) Google output for definitions of terms and searchable text behind figures, and (2) output from external tools used correctly.
ChatGPT 4	The questions were entered in ChatGPT 4.0 as appeared on the exam, similar to the ChatGPT condition, but screenshots of the figures were also uploaded.

In each condition, the chat was prompted to give an answer according to the type of problem (multiple choice and T/F). Since the ChatGPT and ChatGPT4 conditions did not use external tools, only the description of the problem was entered in the chat box for these types of questions. Figures were not utilized in the ChatGPT condition but were utilized in the ChatGPT4 given that ChatGPT 4.0 has the capability to upload and interpret images.

The ChatGPT+ condition used the same chat box as ChatGPT, but the original question was modified as follows to create the chat box input. Each question was first evaluated by a Google search, and any definitions of terms were inserted in the appropriate place. The figures were uploaded to a Google image search, and the results together with the figure captions were added. When external tools were required, the tools were used correctly, and the information generated was also inserted appropriately in the input.

#### 2.2.3 Scoring ChatGPT Output

For all three ChatGPT conditions and all four exams, the output for each individual question was recorded and assessed. The questions that required students to draw for an answer were automatically given no points. Multiple choice and True/False questions were scored as correct/incorrect according to the exam key. Long response questions were scored as if a student had given the answer, according to the completion of the question where key words and concepts had to be present to be awarded full points, and no partial credit was awarded otherwise.

## 3 Results

### 3.1 Assessment of ChatGPT Generated Study Materials

The results of the student survey can be found in Table 3. Students were asked to rank on a Likert scale of 1–7 how useful each of the different learning tools was in helping them prepare for a short quiz on the topic. These included traditional study materials (e.g., lecture notes, text) as well as the materials generated by ChatGPT. In this scale, 1 was not at all useful and 7 was most useful. The average score for each tool is noted along with the standard deviation and the number of students who ranked the tool with a score of 1 or 2 (not at all useful) and 6 or 7 (most useful). Twenty‐one students participated in this survey (greater than 80% of the class).

**TABLE 3:** Student opinions of the perceived usefulness of different study tools scored on a seven‐point Likert scale where 1 was not at all useful and 7 was highly useful.

Learning tool	Avg ± SD	Number of Likert scores of 1 or 2	Number of Likert scores of 6 or 7
Text	6.25 ± 1.21	1	17
Class notes	5.35 ± 0.99	0	7
PowerPoint presentations	4.05 ± 1.90	4	6
Study guides	6.25 ± 1.02	0	19
Electronic HW	3.25 ± 1.16	5	1
Electronic resources/online tools	3.2 ± 1.58	6	1
AI transcript	4.05 ± 2.01	7	6
Chat GPT summary	4.05 ± 1.93	5	5
Chat GPT notes	4.05 ± 1.93	5	5
Chat GPT outline	4.05 ± 1.99	5	6

*Note: N* = 21 students who participated in the survey.

Students found that the text or study guides written by the professor were the most useful in preparing for the quiz, with greater than 80% in one instance and greater than 90% of students in the other valuing these tools as means of preparing for the quiz (average scores of 6.25/7 for both). Only one student found the text to not be of use in preparing for the quiz, while 17 of 21 students found it highly valuable, and 19 of 21 students found professor‐prepared study guides useful.

At the other extreme, students did not value electronic homework or electronic/online resources as helping them prepare for the quiz (average scores of 3.25 and 3.2 out of 7). Nearly a third of students found these tools to be of little use to them in preparing for the quiz, while only one found them highly useful.

Class notes that the students had taken themselves and PowerPoint presentations that were used in class and archived on the class Learning Management System (Blackboard) fell in between these two clusters with average scores of 5.35 for notes and 4.05 for PowerPoint presentations. Likewise, there was increased variability with regard to how individual students felt about these tools. For example, in the instance of PowerPoint presentations, four students found them to be of no use, while seven found them highly useful.

The scores for the Transcript, ChatGPT Summary, Chat GPT Notes, and Chat GPT Outline were nearly identical, with an average score of 4.05 for each (standard deviations varied slightly). Interestingly, scores were also polarized, with nearly a quarter to a third of students finding the tools highly useful and an equal number finding them of little use to them.

### 3.2 Comparison of Chat GPT Exam Performance

**FIGURE 1:**
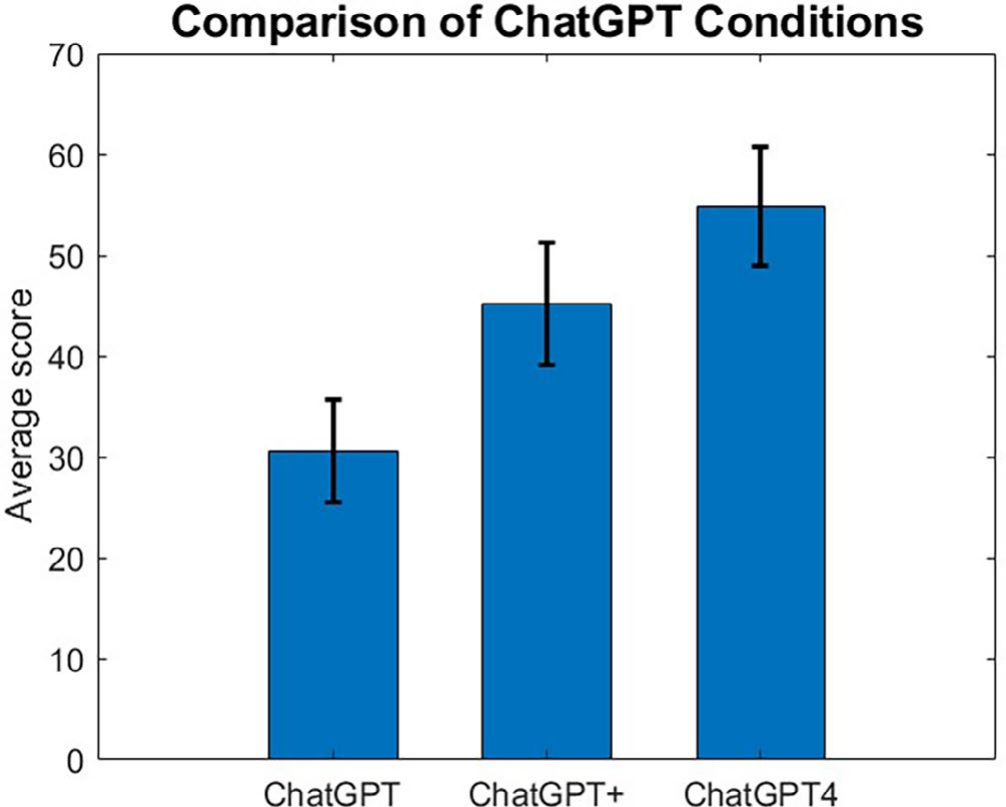
Average scores (percent correct) for the three ChatGPT conditions on the biochemistry exams. The error bars correspond to the standard error of the mean.

We analyzed the scores obtained for ChatGPT across the three conditions (ChatGPT, ChatGPT+ and ChatGPT4). The results show that ChatGPT does not perform well overall. ChatCPT4 performed higher among the three ChatGPT conditions, followed by ChatGPT+, while the ChatGPT condition scored lower. Figure 1 shows the average scores across the three settings. The error bars displayed correspond to the standard error of the mean. There was a statistically significant difference between the means, as determined by a one‐way analysis of variance (ANOVA) (*F*(2, 162) = 4.63, *p* < 0.05). A Tukey–Kramer post hoc test was conducted to determine which conditions were statistically different. The mean score for the ChatGPT condition was significantly different from the mean score for the ChatGPT4 condition.

**FIGURE 2:**
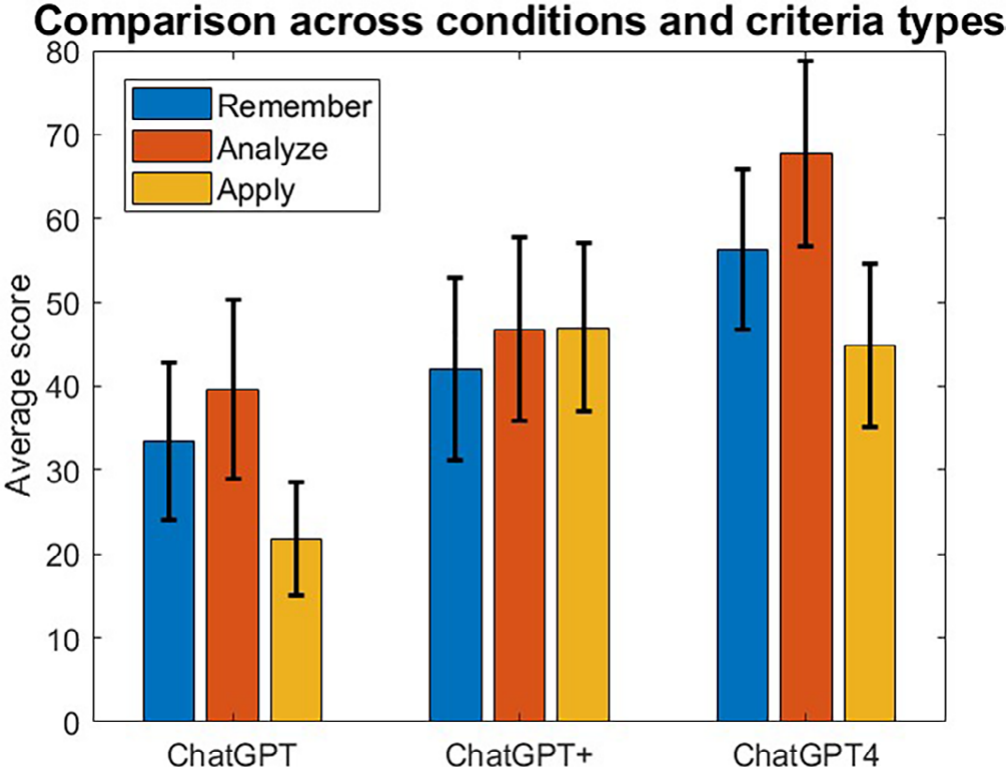
Average scores (percent correct) across conditions and Bloom's Taxonomy categories. The error bars correspond to the standard error of the mean.

We further analyzed the data to investigate the potential effect the type of question has on the exam performance. The analysis shows that overall conditions perform equally well across the types of question classified under Bloom's taxonomy. Figure 2 shows the average scores across the three settings above and the three Bloom's taxonomy criteria: remember, analyze, and apply. The error bars displayed correspond to the standard error of the mean. A two‐way ANOVA revealed that there was no statistically significant interaction between the condition and the criteria factors (*F*(4, 156) = 0.43, *p* = 0.79) and that the Bloom's taxonomy criteria were not a statistically significant factor (*F*(2, 156) = 1.29, *p* = 0.28).

**FIGURE 3:**
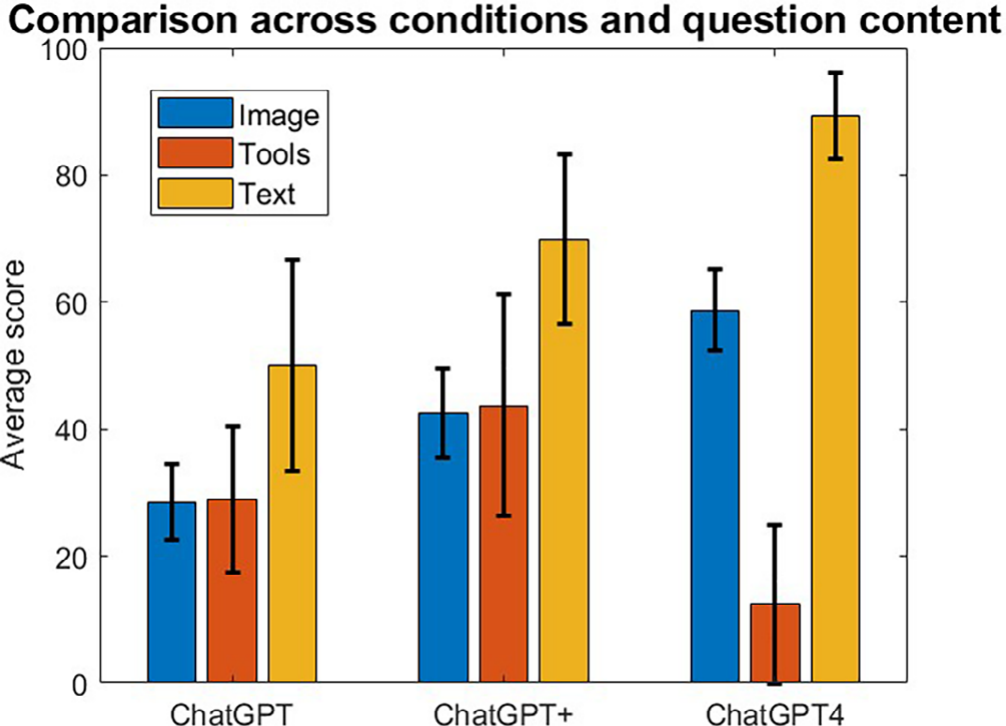
Average scores (percent correct) across conditions and the type of question content. The error bars correspond to the standard error of the mean.

Finally, we analyzed the effect of the question content and found that this factor significantly impacts the performance of ChatGPT. Questions containing text only scored highest across the ChatGPT conditions, while questions containing tools scored lowest. Figure 3 shows the average scores across the three settings above and the three types of question content: image, tools, text. The error bars displayed correspond to the standard error of the mean. A two‐way ANOVA revealed that there was no statistically significant interaction between the condition and the question content (*F*(4, 156) = 1.58, *p* = 0.18) but that the question content was a statistically significant factor (*F*(2, 156) = 4.74, *p* < 0.05). A Tukey–Kramer post hoc test was conducted to determine which mean score subgroups were different. The following statistically significant differences were found: (1) Image‐ChatGPT and Text‐ChatGPT4, (2) Image‐ChatGPT4 and Tools‐ChatGPT4, and (3) Tools‐ChatGPT4 and Text‐ChatGPT4.

## 4 Discussion

In this work, we describe two different applications of artificial intelligence using Chat GPT: generation of study materials for students and assessment of examinations.

Modalities through which students learn are as varied as the students themselves. While it is now appreciated that active learning is a more efficacious way to engage students and prompt learning, as technology improves, we must appreciate that there are multiple ways through which material can be delivered to students and through which they can process content [22, 23]. These include but are not limited to in‐person lectures, videos of lectures or videos on a topic, texts, original research papers, notes, study guides or outlines, or multiple other options. One challenge that students and teachers face is the pairing of learning materials to an individual course. While faculty may often rely more heavily on one source of material over another (e.g., a text or class notes), students may find outside sources that vary in depth of coverage or may emphasize aspects of a topic that are not in alignment with the teacher's plans. Even faculty with a dedicated class plan which they have emphasized for multiple semesters may make small changes to accommodate student questions or changes to the field. By using ChatGPT to generate notes, study guides, and other study materials, we have accomplished two things. First, we have provided materials that were derived from the day's actual experiences, not a historical record. By doing so, we have avoided material that is not on topic or presented at too superficial or too in‐depth a level. Second, we have lowered the barrier for faculty to develop and deliver these materials. By using AI, we have simplified this process and given faculty a significant head start over deriving these tools de novo from their memory or lecture notes.

The current study was conducted in introductory biochemistry courses taught at the junior/senior level, but we believe that some of the conclusions can be generalized to other similar classes. Chat GPT has been used as a teaching tool in different applications in undergraduate medical education, physiology, and microbiology courses [24, 25, 26, 27]. In each of these examples, Chat GPT is being used to answer questions. This aspect of our study uses Chat GPT to summarize and derive materials for student use. This is an underexplored use of the tool, but one that we feel has merit in time savings and provides novel insights into the course material. In this study, Chat GPT accurately summarized the day's lecture but revealed that a disproportionate amount of time was spent on one aspect of beta oxidation (fatty acid transport into mitochondria) over the reactions of beta oxidation. This may be apparent to students when reviewing their notes but was not obvious to the instructor before seeing these results.

A notable difference is that these courses include students who have already developed a relatively larger skill set and are expected to be able to recall and apply concepts from prerequisite coursework. They are also more likely to have developed their own personal study skills. This may alter how students perceive novel learning tools, and it may be different for students in other stages of their academic careers.

In the evaluation of classroom materials, several patterns emerged. The text ranked as one of the most valuable tools that the students used in preparing for the quiz, followed by the study guides written by the professor. It should be disclosed that the professor in this course was also the author of the text used [28]. As known both anecdotally and from prior research, study guides, rubrics, and other support materials are known to be positively perceived by students [29]. Homework and electronic resources ranked lowest in our study. This could be due to the student experience. Homework, for example, while an excellent preparation tool, is not always viewed favorably by students. The AI‐based tools introduced in this study ranked in the middle; however, there was an interesting polarization of these results, with some students finding the generated materials valuable (scores of 6 or 7) while others found them not useful at all (scores of 1 or 2). This polarization could be attributed to differences in study skills, opinions about technology, or simply student preference, but since no additional data was collected, further investigations are required. We did not attempt to assess the usefulness of these materials in this study or correlate them with performance on an assessment; we simply asked students if they used the materials in their studying and if they found them efficacious. The AI‐generated materials were introduced late in the semester, so it is possible there was a bias students had toward materials previously introduced in the course.

Chat GPT performed relatively poorly on examinations with none of the ChatGPT conditions achieving a passing grade (70%). Providing additional information improved, as expected, the performance of ChatGPT 3.5. ChatGPT 4.0 also performed better than ChatGPT 3.5; it seems that there are two factors at play: the ability to handle images as well as increased accuracy overall. This can be seen in our analysis as both the images and text subgroups have higher scores in ChatGPT 4.0 over ChatGPT 3.5.

The type of questions that compose an exam is important; we investigated therefore both the Bloom's taxonomy criteria and the content type (image/tools/text) as potential factors affecting the results [30, 31]. Note that we excluded in our analysis questions that were particularly impossible for ChatGPT, such as questions that require generating drawings or all work conducted with a separate tool. As such, the ChatGPT performance reported in the analysis overestimates the actual ChatGPT performance on the exams. Our data showed that remember, analyze, and apply criteria show almost similar trends with the overall performance across all three settings. The categories show a steady increase across the ChatGPT conditions, with the exception of apply. Question content significantly affected our results, with tools performing the worst, followed by images and best performance in text. However, similar trends applied here too, with increased scores across ChatGPT conditions with the exception of tools, which actually decreased for ChatGPT 4. This explains our earlier observation relating to the apply category, which contained more tools and therefore no increased performance was attained from ChatGPT+ to ChatGPT4. Based on these findings, it would seem that one way to combat ChatGPT usage in exams or homework is to include more tools‐based questions, especially where students work in the tool and information is contained. It is concerning from an academic integrity standpoint that ChatGPT4 performs as well as students in the analyze category.

Others have examined how well Chat GPT is able to perform on examinations in topics ranging from architecture and law to science and medicine [20, 21, 24, 25, 26, 27, 32, 33, 34]. While Chat did not pass our examinations (i.e., attain 70% correct answers) our results were in line with these other studies in several ways. Overall, Chat performed admirably, attaining higher scores on lower‐level Bloom's Taxonomy questions (fact recall) or on multiple choice exams where it could draw from pre‐existing knowledge. It performed less well on higher level Blooms Taxonomy questions where synthesis or creativity is required. Differences between our study and these other studies may reflect differences in what was considered a passing grade on each assessment, how questions were structured (multiple choice vs. free response) as well as the Bloom's level of each question.

We believe that AI stands to be the single biggest benefit and challenge to the current teaching and learning paradigm. For instructors, it can radically streamline the preparation of a course by automating the creation of standard materials: study guides, homework assignments, quizzes, and exams. It may also be used to generate new curriculum, for example, by combining seemingly disparate topics. However, it can undermine academic integrity since students can create the appearance of having mastered the topic even when they did not. This prohibits the teacher from effectively helping those students. For students, AI can be used to create a near‐perfect learning environment, with a 24/7 available artificial tutor and innovative learning materials tailored to the student's ability. Research also shows that students' overreliance on these tools undermines the learning process, and when overutilized for a long period of time, can lead to cognitive decline and increased procrastination [6].

Given students' growing interest in using ChatGPT, we feel that discussions around the current performance of AI on science exams should be made part of the course syllabus; students should be deterred from indiscriminately trusting potentially faulty ChatGPT output. With AI tools developing rapidly, these recommendations will have to be assessed regularly. On the other hand, instructors should be aware that in the real classroom a typical student, even with limited class knowledge, will be able to obtain additional subject information when interacting with ChatGPT and thus will very likely perform over the baseline reported here. Students taking traditional exams with ChatGPT would have an unfair advantage, thus potentially passing exams with less than desired levels of competency in the subject matter.

## 5 Conclusion

We examined the current strengths and weaknesses of an AI (Chat GPT) in several aspects of undergraduate biochemistry courses. Specifically, we examined its ability to generate course material and tested its performance on existing assessments to give guidelines to students and teachers regarding Chat GPT. Overall, Chat GPT performed well in generating study materials and gave mixed results on biochemistry assessments. While AI has great potential to revolutionize education, more work is needed to integrate AI in the classroom to assist both teachers and students.

## Ethics Statement

The first experiment in this study was conducted using institutional research board approval (Otterbein IRB# 23/24–28).

## Conflicts of Interest

The authors declare no conflicts of interest.

## Data Availability

The data that support the findings of this study are available from the corresponding author upon reasonable request.
